# Natural Polymer-Based Hydrogels: From Polymer to Biomedical Applications

**DOI:** 10.3390/pharmaceutics15102514

**Published:** 2023-10-23

**Authors:** Lingling Zhao, Yifan Zhou, Jiaying Zhang, Hongze Liang, Xianwu Chen, Hui Tan

**Affiliations:** 1 School of Materials Science and Chemical Engineering, Ningbo University, Ningbo 315211, China; 2 Center for Child Care and Mental Health (CCCMH), Shenzhen Children’s Hospital, Shenzhen 518038, China; 3 The Affiliated Hospital of Medical School, Ningbo University, Ningbo 315211, China

**Keywords:** hydrogel, natural polymer, drug delivery, tissue engineering, wound healing

## Abstract

Hydrogels prepared from natural polymer have attracted extensive attention in biomedical fields such as drug delivery, wound healing, and regenerative medicine due to their good biocompatibility, degradability, and flexibility. This review outlines the commonly used natural polymer in hydrogel preparation, including cellulose, chitosan, collagen/gelatin, alginate, hyaluronic acid, starch, guar gum, agarose, and dextran. The polymeric structure and process/synthesis of natural polymers are illustrated, and natural polymer-based hydrogels including the hydrogel formation and properties are elaborated. Subsequently, the biomedical applications of hydrogels based on natural polymer in drug delivery, tissue regeneration, wound healing, and other biomedical fields are summarized. Finally, the future perspectives of natural polymers and hydrogels based on them are discussed. For natural polymers, novel technologies such as enzymatic and biological methods have been developed to improve their structural properties, and the development of new natural-based polymers or natural polymer derivatives with high performance is still very important and challenging. For natural polymer-based hydrogels, novel hydrogel materials, like double-network hydrogel, multifunctional composite hydrogels, and hydrogel microrobots have been designed to meet the advanced requirements in biomedical applications, and new strategies such as dual-cross-linking, microfluidic chip, micropatterning, and 3D/4D bioprinting have been explored to fabricate advanced hydrogel materials with designed properties for biomedical applications. Overall, natural polymeric hydrogels have attracted increasing interest in biomedical applications, and the development of novel natural polymer-based materials and new strategies/methods for hydrogel fabrication are highly desirable and still challenging.

## 1. Introduction

Hydrogels are three-dimensional networks formed by hydrophilic polymers through chemical cross-linking (covalent bond, ionic bond) or physical cross-linking (hydrogen bond, van der Waals force, physical entanglement). They can swell in water and retain a certain shape while absorbing a large quantity of water, attracting increased interest in numerous areas since they were first reported by Wichterle and Lim in the 1960s [1]. Natural polymers, which are formed from photosynthesis or a biochemical reaction in the natural world or extracted from natural products, are ideal skeletons for the fabrication of hydrogels because of their diversified properties such as their biocompatibility, biodegradability, and environmental friendliness. Hydrogels based on natural polymers such as alginate, starch, cellulose, chitosan, gelatin, collagen, hyaluronic acid, and so on show a good degradability, biocompatibility, nontoxic degradation products, a good flexibility similar to natural tissue, and are in natural abundance [2], which endow them with widespread applications in medicinal fields, for instance, as carriers for drug delivery, wound dressing for wound healing, substrates for cell culture, scaffolds for tissue regeneration, and so on [3,4].

Biocompatibility is one of the most significant characteristics of hydrogels for biomedical application, referring to the ability of a material to contact with bodily organs with minimum damage to the surrounding tissues and without triggering undesirable immune responses but performing its desired function in a medical treatment [5]. Hydrogels based on natural polymers usually have excellent biocompatibility owing to the intrinsic properties of natural polymers. However, for most of the natural polymeric hydrogels, their limited toughness and poor mechanical properties become major obstacles to their application. Several efforts were made to improve the mechanical properties of hydrogels, including chemical cross-linking, physical cross-linking and mixing with other synthetic/semisynthetic polymers or inorganic particles.

Biodegradability is another essential property of hydrogel materials for biomedical application, such as tissue regeneration, as cells need space to grow, migrate, and proliferate [6]. It refers to the capacity of a substance to break down after interactions with biological elements [7]. Most of the hydrogels prepared from natural polymers can be decomposed under the action of enzymes, allowing cells to spread and reshape their surroundings. The rate of biodegradation is another required factor, which depends on properties such as molecular weight and the amorphous or crystalline and hydrophilic/hydrophobic behaviors of polymers [8].

This review outlines the current progress on natural polymer-based hydrogels in biomedical fields. The most widely used natural polymers such as collagen, gelatin, alginate, cellulose, chitosan, and hyaluronic acid are described (Table 1), and natural polymer-based hydrogels including hydrogels prepared from natural polymers, natural/synthetic polymer, and natural/inorganic particles are summarized. Then, the biomedicine applications of natural polymer-based hydrogels in drug delivery, wound healing, and regenerative medicine are summarized and discussed, respectively. Finally, future perspectives and a conclusion are proposed.

## 2. Natural Polymer-Based Hydrogels

Natural polymers derived from materials in the natural world, such as polysaccharides and proteins, have a good biocompatibility and biodegradability, making them an ideal choice for the fabrication of hydrogels. Polysaccharides used for hydrogel fabrication include cellulose, chitosan, dextran, alginate, hyaluronic acid, as well as their derivatives. There are abundant hydroxyl groups and/or other functional groups (amino, carboxyl groups, and so on) on the chains of polysaccharides, offering versatile opportunities to prepare polymer-based hydrogels via chemical or physical cross-linking. Proteins including collagen, gelatin, and fibrin are essentially polymers of amino acids. It is thought that proteins can form fibrils in nanometer width and micrometer length via intermolecular and/or intramolecular forces (such as hydrogen bonds, electrostatic interactions, and hydrophobic effects) and further form three-dimensional hydrogels via self-organization and entangling under appropriate conditions [20].

### 2.1. Cellulose and Cellulose-Based Hydrogels

**Figure 1:**
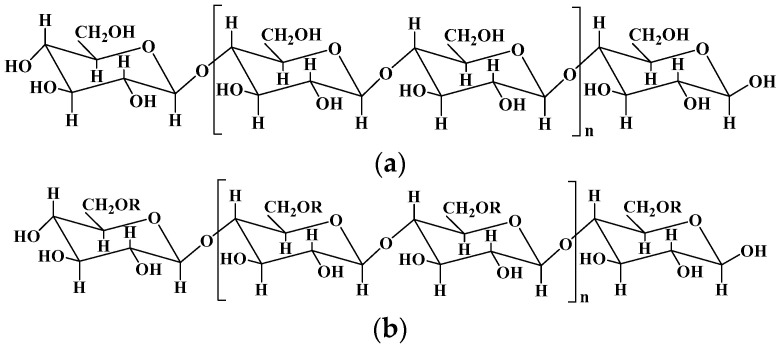
The chemical structures of cellulose (**a**) and some important derivatives of cellulose (**b**).

Cellulose is the most abundant natural polymer compound on earth, consisting of β (1-4)-glycosidic-linked glucose units (Figure 1). Cellulose organizes in a rather intricate supramolecular structure formed by the intermolecular cohesion of cellulose molecules, which is an extended intra/intermolecular network of hydrogen bonds. In the dissolution process of cellulose, the intramolecular hydrogen bonds are broken, and the supramolecular structure of cellulose is disintegrated, enhancing the activity of hydroxyl in cellulose, and making it easy to combine with other natural or synthetic polymers by reconstructing hydrogen bonds. Therefore, cellulose is one of the ideal candidates for hydrogels’ preparation, endowing cellulose composite hydrogels with specific performance, such as biodegradability, renewability, flexibility, and high mechanical strength [21].

Methylcellulose: R = -H, -CH_3_; ethylcellulose: R = -H, -CH_2_CH_3_; hydroxyethyl cellulose: R = -H, -CH_2_CH_2_OH; carboxymethylcellulose: R = -H, -CH_2_COOH; hydroxypropyl cellulose: R = -H, -CH_2_CH(OH)CH_3_; hydroxy propyl methyl cellulose: R = -H, -CH_3_, CH_2_CH(OH)CH_3_.

Cellulose can be divided into plant cellulose and bacterial cellulose. Plant cellulose is widely sourced from cotton, wood, and other plants, such as cardamom fiber, seed fiber, and wood fiber, and is the most abundant organic substance in nature [22]. Plant cellulose is mainly produced in a nonpure form as lignocellulose, and can be purified using an ecofriendly biological method and a non-ecofriendly chemical method [23]. The biological method depends on microbial enzymes and has a low productivity, while the chemical method has many steps and types of equipment and a high productivity. Compared with plant cellulose, bacterial cellulose produced via certain types of bacteria has a high purity and functionality, which is a major alternative source of plant cellulose [24]. Bacterial cellulose can be produced by the biosynthesis of certain types of Gram-negative and Gram-positive strains in high-glucose-containing media.

Nanocellulose (NC) is a mesoscopic material formed in the regeneration process of cellulose, which combines the advantages of both cellulose and nanomaterials. NC, including cellulose nanocrystals (CNCs) and cellulose nanofibrils (CNFs), has been widely used in the field of functional materials due to its unique morphology of nanostructures and featuring many advantages like excellent mechanical properties, biodegradability, and environmental friendliness [25,26]. For example, CNFs prepared by TEMPO (2, 2, 6, 6-Tetramethylpiperidine-1-oxyl)-mediated oxidation has plenty of hydroxyl groups and carboxyl groups on their surface, endowing CNFs with a good stability and dispersibility without aggregation in water, and making them easy to combine with other polymers or nanoparticles to construct novel reinforced composite materials and hydrogels [27,28].

The fabrication of cellulose-based hydrogels is challenging because cellulose is hardly soluble in common solvents due to its highly extended hydrogen-bonded structure. One of the strategies to resolve this issue is the development of several solvent systems, for example dimethylacetamide, alkali/urea (or thiourea) aqueous systems, and ionic liquids [29,30]. For instance, several solvent systems including LiCl/dimethylacetamide, paraformaldehyde/dimethyl sulfoxide, and triethylammonium chloride/dimethyl sulfoxide have been used to directly fabricate cellulose hydrogels [31], and some hydrophilic ionic liquids, such as 1-butyl-3-methylimidazolium chloride and 1-allyl-3-methylimidazolium chloride, have been used to dissolve cellulose [32]. Chemical modification is another effective method to resolve the poor solubility of cellulose. Hydroxyl and carboxyl groups are the most commonly used active groups introduced into the skeleton of cellulose, obtaining various cellulose derivatives including carboxymethyl cellulose, hydroxyethyl cellulose, hydroxypropyl cellulose, and hydroxypropyl methyl cellulose [33,34]. Cellulose-based hydrogels can be formed via the cross-linking between these functional groups, for instance, the esterification of hydroxyl and carboxyl groups by carbodiimide-condensing agents, Michael’s addition reaction between hydroxyl groups and carbon–carbon double bonds under alkaline conditions, epoxide and alkyl halide cross-linking under strong basic and high-temperature conditions, and free radical polymerization [22,35].

Cellulose–gelatin hydrogels with high strength and pH-responsiveness were prepared using the cyclic freezing–thawing method [36]. The repeated freezing–thawing cycles played a vital role in the formation of the supramolecular network structure via physical cross-linking between cellulose and gelatin. The superior mechanical performance contributed to the combination effect of the hydrogen bond and the reinforcement of CNFs. A bacterial cellulose-based hydrogel with good mechanical properties and improved ionic conductivity was prepared for thermo-electrochemical cells in practical wearable electronics by introducing highly soluble urea and the thermodiffusion effect of NaCl [37]. The bacterial cellulose hydrogel-based electrolyte had a nanofiber-porous 3D network structure, and its nanochannels contained plenty of surface hydroxyl groups, which favored the transport of positive ions.

### 2.2. Chitosan and Chitosan-Based Hydrogels

**Figure 2:**
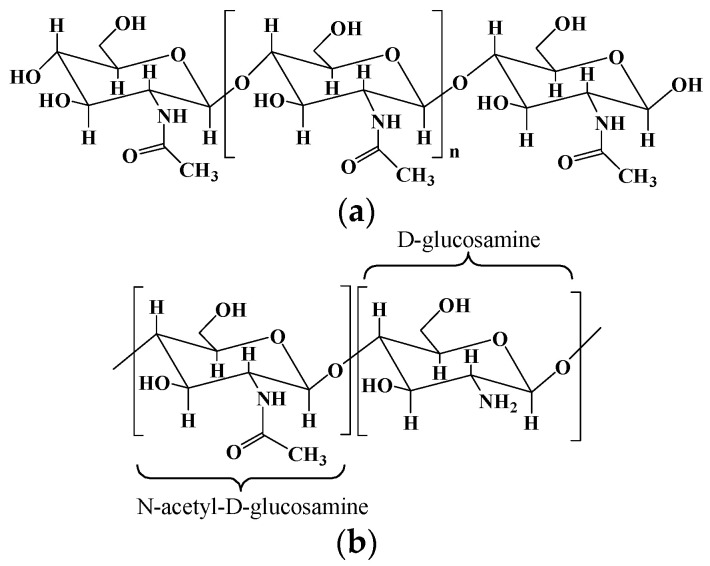
The chemical structures of chitin (**a**) and chitosan (**b**).

Chitosan is a natural polycationic polymer with hydrophilic properties, consisting of the repeating residues of D-glucosamine and N-acetyl-D-glucosamine (Figure 2) [10]. It is obtained by the partial deacetylation of chitin, one of the most abundant polymers after cellulose, extracted from the fungal cell walls and the exoskeleton of crustaceans/insect [38]. Generally, chitosan should have a deacetylation degree of 60, containing at least 60% of D-glucosamine for the deacetylated chitin [10]. Chitosan is an analogous of glucosaminoglycan, one of the main components found in the extracellular matrix (ECM) of some living tissues, so chitosan can be used to mimic the ECM in regenerative medicine and has been widely studied in the areas of biotechnology [39,40]. As the only cationic polysaccharide found in natural polysaccharides to date, chitosan is well known for its antimicrobial and antifungal properties. It is thought that the positively charged chitosan can interact with the negatively charged surfaces of cells and microbes, thereby inhibiting the absorption and excretion of substances [41]. Chitosan exhibits a degradability in vivo by human protease such as lysozyme due to its multiple amino groups [42]. Recently, studies have highlighted the antitumor activity, mucoadhesive, and hemostatic properties of chitosan, which render its applications in biomedicine and pharmaceuticals promising. What is more, due to the abundant hydroxyl and amine groups on the chitosan backbone, its chemical properties can be further tailored by adding functionalities to develop functionalized chitosan for biomedical applications.

The two main sources of chitosan are animal sources (crustaceans and fungal mycelia) with seasonal supplies and mushroom sources with a better reproducibility. Chitosan is derived from the partial deacetylation of chitin using a chemical or biological approach. The chemical hydrolysis is performed under severe alkaline conditions (i.e., concentrated sodium hydroxide and sodium borohydride), and the biological method is performed by particular bacteria and/or enzymes [11]. 

Chitosan-based hydrogels are formed by physical association or chemical cross-linking [43]. In physically cross-linked hydrogels, non-covalent interactions, namely, electrostatic interactions, hydrophobic interactions, and hydrogen bonding between polymer chains, are used to fabricate the gel network. In chemically cross-linked hydrogels, cross-linker agents, secondary polymerizations, click chemistry, or irradiation chemistry is used to form the chitosan-based hydrogels. Many bifunctional/polyfunctional molecules with aldehyde, including glutaraldehyde, glycidyl ether, isocyanate, acrylate, azides, and so on, have been used to cross-link chitosan polymers and fabricate chitosan-based hydrogels, especially hydrogels with environmental sensitivity [44,45]. Chitosan hydrogels with self-healing ability were prepared using natural vanillin as a cross-linking agent [46]. The hydrogel network contained hybrid linkages of Schiff base bonds and hydrogen bonds. The concentration of vanillin has an important effect on the self-healing ability of the hydrogel because the self-healing ability mainly comes from the reconstruction of the dynamic Schiff base bond. Since chitosan itself has poor solubility, chemical modification can be used to produce chitosan derivatives with good water solubility. Succinylation is a simple and effective way to improve the water solubility of chitosan and obtain new functional groups, and succinylated chitosan can be ionically cross-linked with glucose-6-phosphate to create a pH-sensitive hydrogel [47]. The succinylated chitosan hydrogel produces the rapid gelation and efficient release of drug, and the drug release can be controlled by adjusting the succinylation and temperature, suggesting a bright and promising future for cancer and inflammation treatments. Ion-cross-linked succinylated chitosan hydrogels via glucose-6-phosphate were prepared as bone graft material carriers for the repair of bone defects [48]. The succinylated chitosan hydrogels had a high decomposition rate and good biocompatibility, could hold bone graft materials in the transplantation area, and showed a high cell-growth rate and bone differentiation rate.

Chitosan-based hydrogels have good biocompatibility, biodegradability, and antimicrobial properties and are ideal candidates for drug delivery and tissue engineering. However, the applications of chitosan scaffolds in tissue regeneration have been limited due to their insufficient mechanical strength and inadequate degradation rate, so the composite scaffolds prepared by mixing chitosan with other functional substances will have better tissue regeneration effect [49]. Liu et al. reported a novel type of homogeneously structured polyelectrolyte complexes (PEC) hydrogel with electro-responsive performance and high mechanical strength based on chitosan and carboxymethylcellulose using the cyclic freezing–thawing method [50] The PEC hydrogel underwent a variety of programmable 3D shape transformations, such as helix, flower, V- and M-like shapes and other intermediate variations owing to the asymmetric deformation of gel strips caused by the uneven osmotic stress on both sides of the hydrogel. 

### 2.3. Collagen/Gelatin and Collagen/Gelatin-Based Hydrogel

**Figure 3:**
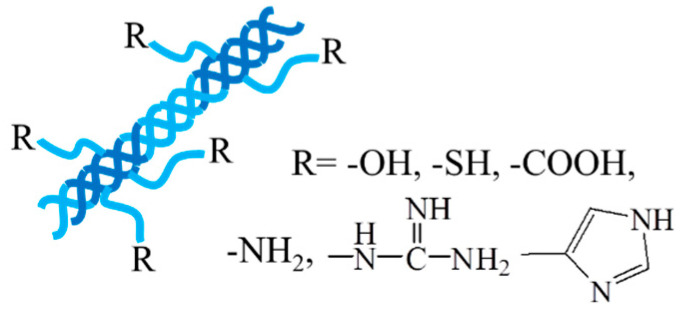
The triple-helical fibrous structure of collagen.

Collagen, as one of the most abundant renewable natural polymers along with cellulose and chitosan, has significant applications in the biomedical field [51,52]. It plays an important role in structural proteins for most tissues, i.e., skin, bones, muscles, blood vessels, and cartilages, and contains plenty of functional groups, i.e., hydroxyl, amino, carboxyl, guanidyl, and imidazoles, endowing collagen with many physical and chemical properties. Collagen is able to self-assemble into a triple-helical fibrous structure under physiological conditions (Figure 3), giving collagen a great tensile strength and durability [53]. Collagen has many excellent natural characteristics, such as hydrophilicity, biocompatibility, biodegradability, nonimmunogenicity, and mechanical durability, making it an essential component in investigations in the biomedical field [54]. For instance, in engineered tissues, collagen can provide mechanical strength to other amorphous hydrogels and modulate the hydrogel to mimic native tissues, providing critical recognition sites for cellular migration and attachment and long-term structural support for tissues [55]. In addition, degradability is another crucial factor to be considered in tissue engineering applications. Due to the collagenase activity in the body, the intrinsic degradability of collagen enables collagen-based hydrogels to have a bright application prospect in the biomedical field including smart drug delivery and tissue regeneration.

Animal is the main source of collagen, and the animal-derived collagen can be obtained from porcine skin, bovine tendon, rat tail, or marine sources by extraction and purification using a chemical or enzyme method. The commonly used agents for the chemical treatment include a neutral salt solution, an alkali solution, dilute acetic acid, and hydrochloric acid. Moreover, recombinant collagen produced by recombinant technology and biosynthesis is an alternative source of collagen, which expressed in yeast, *Escherichia coli*, mammalian cells, insect cells, tobacco plants, corn seeds, and so on [12].

The chemical modification of collagen is generally applied to create desired properties since the direct usage of collagen in specific applications may sometimes lead to several problems like calcium deposition, high thrombogenicity, uncontrollable degradation rate, inadequate mechanical properties, and so on. The chemical modification can be achieved through structural decoration by the insertion of new functional groups and/or by the combination of novel materials containing new functional groups [51,56]. Hydrogels with M2 macrophage-polarization and anti-inflammatory properties were prepared through enzymatic cross-linking of tyramine-grafted collagen and gallate dimer-grafted hyaluronic acid [57]. The hydrogels integrated with deferoxamine-loaded mesoporous polydopamine nanoparticles have enhanced mechanical strength and desirable tissue adhesion and injectability, showing an improved repair effect of diabetic wounds.

Gelatin is a denatured, water-soluble polypeptide derived from collagen being irreversibly hydrolyzed [58]. The hydrolysis of collagen dissociates the triple helix into three peptide chains and decomposes collagen to gelatin, making the material biocompatible and biodegradable for cell growth [59]. Gelatin aqueous solution at a concentration of 0.5–50 wt% is in a sol state above the melting temperature (31.7–34.2 °C) and forms a thermoreversible gel after cooling [60]. However, the low melting temperature limits its application under physiological conditions. The addition of salt or other small soluble compounds could lead to a structural reorganization of the gelatin hydrogels due to the change in the interactions between gelatin molecules, such as hydrogen bonding, hydrophobic forces, and electrostatic forces [61,62]. Gelatin methacrylate (GelMA) is a photo cross-linkable gelatin derivative prepared by modifying the reactive side groups of gelatin using glycidyl methacrylate and has received attention in biological fields [63,64]. GelMA can be cross-linked to form hydrogel via photo-polymerization due to the presence of methacrylate, and the stiffness and porosity of the GelMA hydrogel can be regulated by controlling the production parameters such as hydrogel concentration, degree of functionalization, UV intensity, and additive supplementation [65].

### 2.4. Alginate and Alginate-Based Hydrogel

**Figure 4:**
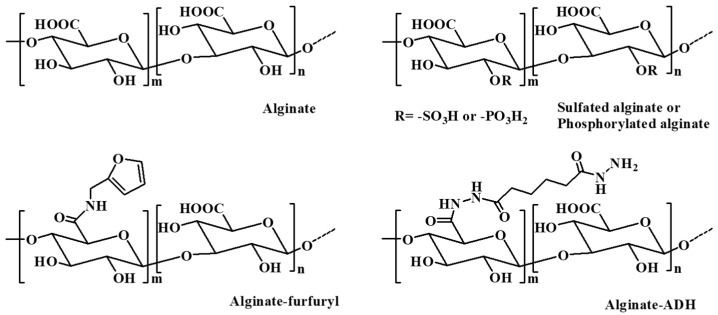
The chemical structure of alginate and some alginate derivatives.

Alginate is a commonly available natural biopolymer, which is a linear anionic polysaccharide consisting of repeated residues of α-L glucuronate (G) and β-D mannuronate (M) (Figure 4) [66]. It provides biocompatibility, biodegradability, non-antigenicity, chelating ability as well as a good stability for a long time [67]. Alginate can absorb large quantities of biological liquids and be purified to prevent immunogenicity, rendering alginate an ideal polymer for hydrogel preparation under mild conditions [68].

Alginate can be extracted from brown algae using alkali solutions (typically a sodium hydroxide solution). The extraction liquid is filtered, and then alginate is precipitated by adding sodium or calcium to the filtrate. In addition, bacterial alginate produced by bacterial biosynthesis from Azotobacter and Pseudomonas is another source of alginate, which provides alginate with more defined chemical structures and physical properties than that of seaweed-derived alginate [13]. 

Naturally obtained alginate may contain various impurities, including heavy metals, proteins, and endotoxins. These impurities can affect its application in the field of pharmaceuticals, especially for parenteral administration. Alginate can be obtained in an ultrapure form by a multistep extraction procedure, which has a controlled pyrogenicity and is suitable for implants in combination with drugs [69,70]. It has been reported that ultrapure alginate is effective in hemostasis, antiadhesion, and wound healing. Ultrapure alginate bilayer sponges were prepared by cross-linking two distinct ultrapure alginates with different molecular weight with calcium ions and lyophilized [71]. The bilayer sponges reduced adhesion by nearly 38% compared to the control group in the Pean crush hepatectomy model in rats. Simultaneously, by covering trauma with endotoxin-free sponges, the ultrapure alginate promoted the regeneration of mesothelium and supported wound healing. Further, a compression process was used to regulate the thickness and improve the mechanical property of ultrapure alginate bilayer sponges after preparing the initial sponges through lyophilization [72]. The results showed that the compressed ultrapure alginate bilayer sponges with the optimum thickness (100 μm) could balance the antiperitoneal adhesion and hemostasis simultaneously.

Alginate hydrogels can maintain a structural similarity to the ECM in tissues and be administrated to play several vital roles; thus, these hydrogels have attracted remarkable interest in biomedical applications, such as wound repair, drug delivery, and tissue regeneration [66]. Various approaches have been applied to prepare alginate-based hydrogels, including ionic cross-linking with divalent cations (i.e., Ba^2+^, Fe^3+^, Ca^2+^) [73,74], covalent cross-linking with multifunctional molecules (i.e., poly(acrylamide-co-hydrazide, poly(ethylene glycol)-diamines)) [75,76], and thermal gelation and in situ copolymerization of thermosensitive polymers (i.e., N-isopropylacrylamide (NIPAAm)) by UV irradiation [77]. In addition, various alginate derivatives including amphiphilic alginate and cell-interactive alginate have also been used in the fabrication of hydrogels for biomedical applications. Amphiphilic alginate derivatives obtained by coupling hydrophobic moieties, such as alkyl chains and hydrophobic polymers, to an alginate skeleton can self-assemble to form hydrogels in aqueous solution, which have promising prospect in drug delivery systems [78].

The mechanical properties of alginate hydrogels formed through intramolecular cross-linking via divalent cations are directly related to the concentration of the cross-linking cation, the molecular weight and length of the G-blocks [79]. Alginate hydrogels prepared with high cation concentrations, a high molecular weight, and a high G-content alginate have a relatively high stiffness [80]. The mechanical properties of alginate hydrogels significantly control the stability of the gel and thus affect their biomedical applications, including the drug release rate from the gel, as well as the phenotype and function of the cells encapsulated in the alginate gels [13].

Ionically cross-linked alginate hydrogels can be dissolved by releasing the divalent ions cross-linking agent into the surrounding medium. However, due to the lack of corresponding enzymes in mammals, such as alginase, and the fact that the average molecular weight of many commercially available alginates is higher than the clearance threshold of the kidneys, alginate cannot be completely cleared from the body [81]. The partial oxidation of alginate chains is an attractive method to make alginate degradable in physiological conditions. The slight oxidization of alginate alters the chain conformation to an open-chain adduct, enabling the degradation of the alginate backbone [13]. In the presence of divalent cations, the partial oxidation of alginate does not significantly influence its gel-forming ability, and the degradation rate of the resultant hydrogels is largely dependent on the degree of oxidation, the pH, and the temperature of the medium [82].

In situ cross-linked alginate hydrogels with calcium ions were designed for myocardial infarction repair [83,84]. A partially cross-linked alginate solution injected into the infarct zone underwent a rapid gelation and phase transition to hydrogel because of the elevated calcium concentrations at the acute infarct site. The calcium cross-linked alginate hydrogel degraded within six weeks after administration via the ion exchange between cross-linking calcium ions and sodium ions from the surrounding tissue. Beneficial therapeutic effects of the calcium cross-linked alginate hydrogels were observed in rat and pig models of acute myocardial infarction, where the hydrogels were replaced by host tissues composed of myofibroblasts and blood capillaries. Hydrogels prepared by cell-interactive alginate, which was synthesized by chemically coupling cell-adhesive peptides (i.e., RGD) as side-chains, are crucial for cell growth and tissue regeneration due to the promotion and regulation of cellular interactions [85]. Hong et al. developed a polymeric hydrogel based on an alginate–boronic acid conjugate through borate ester cross-linking between the intrinsic cis-diol at the alginate backbones and boronic acid [86]. The prepared hydrogel demonstrated unprecedented multifunctionalities simultaneously, such as self-healing capacity, stretchability, shear-thinning, stimuli sensitivity, and adhesive and reshaping properties, which were owing to the reversible intermolecular/intramolecular interactions resulting from the dynamic complexation and dissociation of the borate ester.

### 2.5. Hyaluronic Acid and Hyaluronic Acid-Based Hydrogel

**Figure 5:**
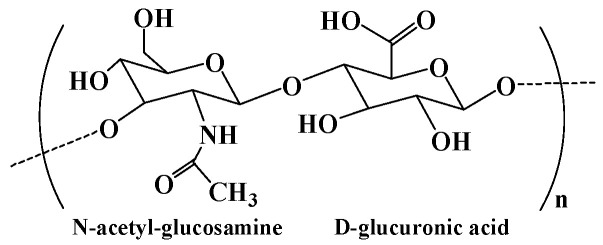
The chemical structure of hyaluronic acid.

Hyaluronic acid is a natural polysaccharide found in the ECM prominently throughout the body, comprising N-acetyl-glucosamine and D-glucuronic acid (Figure 5) residues [87]. Hyaluronic acid is well known for its bioactivity, which is the main nonsulfuric glycosaminoglycan that regulates many cellular responses in the ECM [2,88]. As a main component of the ECM, hyaluronic acid plays a significant role in many physiological functions, including lubrication, water absorption, and retention for tissue and the ECM, and structural and space-filling functions, and interacts with various cell receptors to coordinate cell communication and behavior [89]. Because of its nontoxicity, nonallergy, biocompatibility, and biodegradability, hyaluronic acid has been widely used as biomedical materials including tissue engineering scaffolds, wound dressings, and drug carriers [90,91]. Hyaluronic acid degrades rapidly in the body and is often used in combination with other materials. For instance, the chemical modification and cross-linking of hyaluronic acid can extend the retention time in vivo [2]. The chemical modification of hyaluronic acid is mainly focused on three distinct functional groups: the glucuronic carboxylic acids, the primary and secondary hydroxyl groups, and the N-acetyl groups. Altering any of these functional groups may result in changes in the mechanical properties, chemical properties, and biological activity of subsequent hyaluronic acid biomaterials [91]. In addition, hyaluronic acid hydrogel can be formed by the auto-cross-linking of intra- and intermolecular ester bonds between the reaction of carboxyl groups and hydroxyl groups. The auto-cross-linking improves the viscoelasticity of hydrogels, and this property can be tuned by changing the esterification reaction conditions [70].

Hyaluronic acid is mainly manufactured via extraction from animal tissues (including human umbilical cords, vitreous humor of cattle, bovine synovial fluid, and rooster combs) and from microbial/bacterial fermentation using pathogenic bacteria and nonpathogenic bacteria [14]. Recently, a novel enzymatic technology for hyaluronic acid manufacture has been developed, and hyaluronic acid with a high molecular weight, defined chain length, and low polydispersity can be enzymatic polymerized using UDP-sugar monomers [92].

Hyaluronic acid contains hydroxyl and carboxyl functional groups in the main chain, which can be chemically modified to obtain hyaluronic acid derivatives with unique biological and physicochemical properties. The carboxyl group can be modified to synthesize hyaluronic acid derivatives with ester (by alkyl halides, diazomethane, and tosylate activation) and amide (using carbodiimides or carbonyldiimidazole) [90]. For example, adipic dihydrazide functionalized hyaluronic acid (HA-ADH) was obtained by coupling adipic dihydrazide to hyaluronic acid through the amidation between the carboxyl group of hyaluronic acid and the amine group of adipic dihydrazide. Hydrogels integrated with sanguinarine-loaded gelatin microspheres were prepared based on HA-ADH and oxidized dextran through Schiff’s base cross-linking for wound healing, exhibiting improved re-epithelialization and ECM remodeling, enhanced antibacterial activities, and decreased inflammatory responses [93]. In addition, the vicinal diol in hyaluronic acid can be oxidized to aldehyde groups by sodium periodate to form aldehyde–hyaluronic acid, and chitosan–hyaluronic acid hydrogel was formed through Schiff’s base cross-linking for the immobilization of insulin-like growth factor 1, which improved stem cell therapy for limb ischemia [94]. Moreover, hyaluronidase can promote the decomposition of hyaluronic acid, and its concentration was found to be remarkably higher in various cancerous cells [95]. Thus, hyaluronic acid-based hydrogels are commonly degradable and can be used as a local therapy carrier. In situ-forming hyaluronic acid-based hydrogel was prepared as a carrier to locally deliver adipose-derived stem cells (ASCs) into the salivary gland [96]. The results showed that the retention of locally delivered ASCs by the hyaluronic acid-based hydrogel could enhance the paracrine effect of ASCs and provide a more efficient alleviation of radiation-induced salivary gland damage.

### 2.6. Starch and Starch-Based Hydrogel

**Figure 6:**
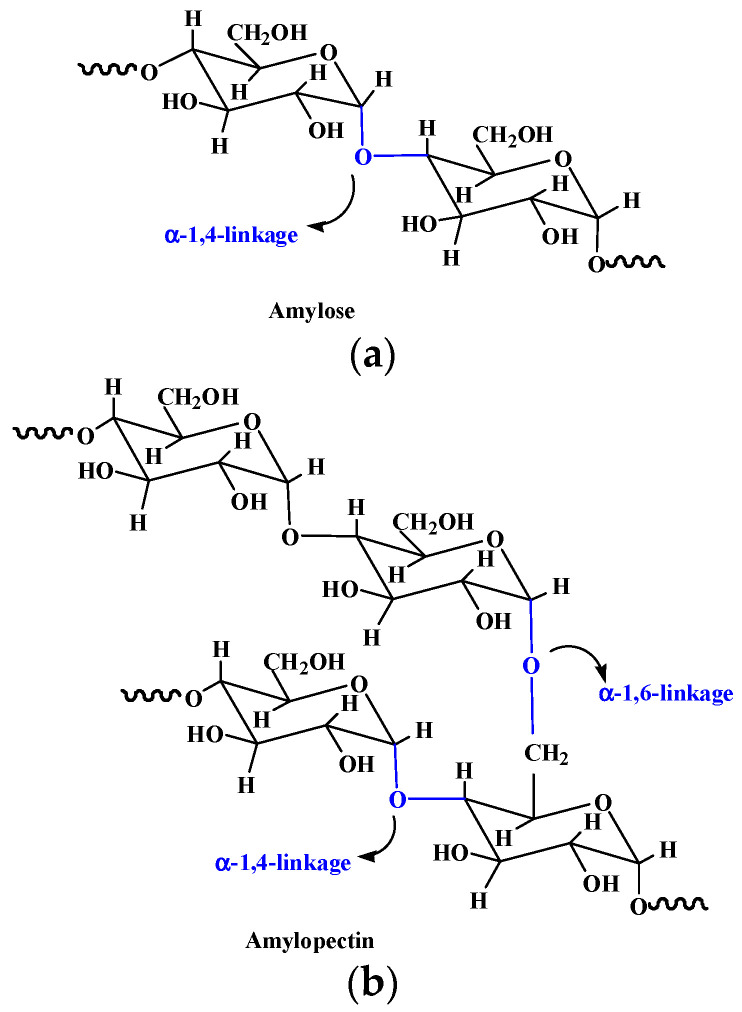
Chemical structures of amylose (**a**) and amylopectin (**b**) in starch.

Starch is a natural polysaccharide composed of glucose repeating units through α-D-(1-4) and α-D-(1-6)-glycosidic linkage (Figure 6) [97,98]. It has a wide range of applications in the food, agriculture, biomedical, and pharmaceutical fields due to its low cost, renewability, biodegradability, and biocompatibility. Starch exhibits a granular appearance in nature, which is called starch granule, containing a small number of proteins, fatty acids, and minerals [15]. Amylose and amylopectin represent the primary two types of polysaccharides in the starch structure. Amylose with a linear structure is known for the double-helix formation due to its left-handed helical conformation and can form supramolecular inclusion complexes with guest molecules [99]. Amylopectin has a more highly branched structure than that of amylose and can form a helical and crystallized structure, playing an important role in stabilizing starch granules’ structure [100]. Variations in amylose and amylopectin have a great influence on the gelatinization and degradation properties of starches, and in turn, play a substantial role in starch hydrogels’ elaboration. Hydrogels produced by low amylose starches are partially or completely water-soluble, while those produced by high amylose starches have a higher structural integrity [101]. Containing plenty of hydroxyl groups, starch has excellent hydrophilic properties and is an ideal candidate for the development of hydrogels with a higher swelling capacity and enhanced biodegradation properties [102]. Compared to native ones, modified starch is a superior material for preparing hydrogels with better properties. For instance, ozonated cassava starch hydrogels have good pasting properties and printability, and an ozonization for 30 min can improve the performance of hydrogels, especially their printability [103].

Starch can be extracted from seeds (such as beans, peas, grasses, cereal grains, etc.), roots, tubers, stems, fruits, and all leaves. The component of amylose and amylopectin in starch varies depending on the plant source. For instance, cornstarch contains about 28 wt% of amylose, and amylose in cassava starches is about 17 wt%, while amylose in waxy potato starches is only 8 wt% [104].

Starch-based hydrogels can be formed by environmentally and friendly physical methods including starch retrogradation and extrusion [15]. The formation of starch hydrogels by retrogradation is due to the rearrangement of amylose during the heating–cooling process. In addition, various chemical methods including etherification and grafting can be adopted to fabricate starch-based hydrogels [98]. The hydroxy groups in the starch structure can be substituted by various ether groups like carboxy-methyl to form etherified starches, and different vinyl monomers (such as acrylamide, acrylic acid, and so on) can be grafted on starch in the grafting method [15]. Light, curable, starch-based hydrogels with Fe_3_O_4_ nanoparticles encapsulation were prepared by cross-linking of acrylic-glycol modified starch under blue light, exhibiting a controllable release of quercetin, improved bioavailability, and enhanced mechanical properties [105].

### 2.7. Guar Gum and Guar Gum-Based Hydrogel

**Figure 7:**
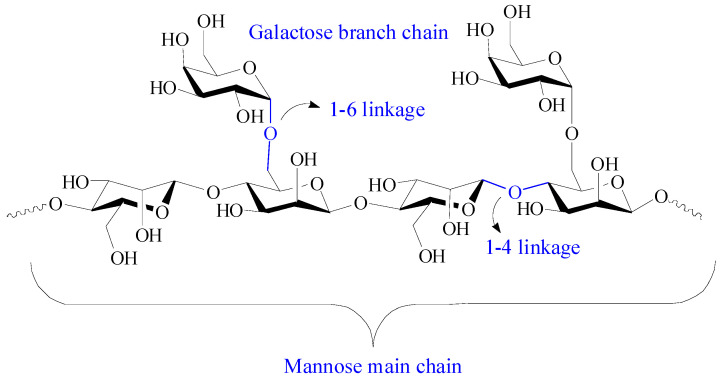
Chemical structure of guar gum.

Guar gum is a nonionic natural polysaccharide isolated from the seed of leguminous plant called *Cyamopsis tetragonoloba*. It comprises linear main chains of (1-4)-β-*D*-mannopyranosyl units and branch chains of α-*D*-galactpyanosyl units connected by (1-6) linkages (Figure 7) [16]. Guar gum has attracted great attention in the biomedical field due to its hydrophilicity, nontoxicity, easy availability, biocompatibility, and biodegradability. Guar gum contains primary and secondary hydroxyl groups, and its physicochemical and biological properties can be adjusted by chemical modification or grafting with other functional materials to suit desired biomedical applications. Guar gum-based biomaterials have aroused great interest in drug delivery systems due to their enhanced drug loading, sustained drug delivery behavior, microbial degradability, improved firmness over a wide pH range, and pH-dependent hydration [106].

With abundant hydroxyl groups, guar gum can form adhesive and stimuli-responsive hydrogels with borate by chemical cross-linking of borate–diol interactions [107]. However, due to the reversibility of the borate ester bond, neat guar gum-based hydrogel has an instability and low strength which restrain its wide application. In addition, galactomannan is sensitive to temperature-based degradation, and the rheological value of a guar gum solution decreases with the storage time, indicating the degradation of polymers [108,109]. In fact, the mechanical strength of borate-based guar gum hydrogel showed a change between 25 °C and 37 °C, which is an unsatisfactory property for wound healing and cellular scaffolds [110]. An attractive approach to overcome this deficiency is the involvement of other polymers, such as alginate and polyacrylic acid, to form dual-network hydrogels [111,112].

pH responsive and self-healable guar gum–borate hydrogels were developed for muco-adhesion [113]. The results of burst pressure tests showed that 1% *w/v* guar gum-borated hydrogel could resist an 8 ± 2 kPa pressure at pH 7.4, which was comparable to fibrin glue and higher than that at solution (pH 5) and brittle gel (pH 10) conditions. Guar gum-based hydrogel with good self-healing capacity, injectability, and adhesion was developed by the introduction of the gel-polydopamine complex and CNCs [109]. Dopamine self-polymerizes to polydopamine on gelatin chains under alkaline condition, and gelatin–polydopamine is further cross-linked to guar gum and CNC via a borate–diol bond, intramolecular Schiff base, and Michael’s addition reaction. CNC also acts as a reinforcer to contribute mechanical strength to the hydrogel. The obtained guar gum-based hydrogels have excellent cytocompatibility and hemolysis ratio, suggesting broad prospects in the biomedical field.

### 2.8. Agarose and Agarose-Based Hydrogel

**Figure 8:**
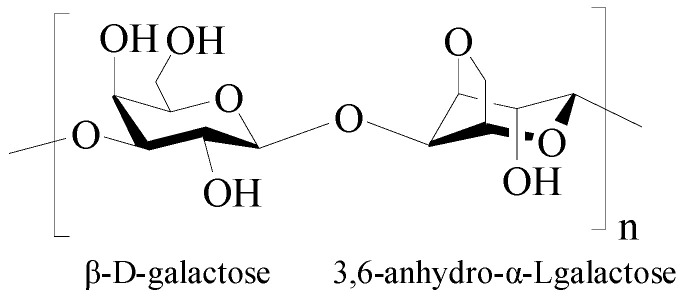
Chemical structure of agarose.

Agarose is a natural oxygen-rich polysaccharide extracted from seaweed, consisting of alternating 1,3-linked β-D-galactose and 1,4-linked 3,6-anhydro-α-Lgalactose units (Figure 8) [17]. Agarose has good biodegradability, biocompatibility, antibacterial properties, perfect cell/matrix interaction, and high hydrophilicity and is widely used in biomedicine and bioengineering. Agarose has an effective porosity with interconnected pores and channels, facilitating the transportation of the material. Moreover, the uniform pore-size distribution and the compatibility of the porous architecture with tissues/organs endow agarose materials with a high ability for controlled drug release [114,115]. A notable feature of agarose is its self-gelling behavior at low temperatures, where the polysaccharide forms a double-helix structure, creating a water-insoluble, gel-type structure, and the melting temperature of agarose gel ranges 80–90 °C [116]. The agarose gel strength is related to the content of 3,6-anhydrogalactose in agarose, and the higher the content, the greater the gel strength [117]. However, the application of agarose gels has been limited due to its high gelling temperature and high rigidity [118]. The high gelling temperature of agarose would inactivate the heat-sensitive tissues or drugs during the gelling process. One effective method to lowering the gelling temperature of the agarose is to introduce functional groups into agarose to hinder the formation of a helical structure at low temperatures, such as agarose modification through acetylation, alkylation, alkenylation, acylation, and oxyalkylation [119]. In addition, agarose can form a stable gel even when other biopolymers are added to the matrix, so by mixing different biopolymers as regulators, agarose has the ability to form stable hybrid hydrogels with tunable selective profiles [120].

β-cyclodextrin functionalized agarose (CFA) gel with low gelling temperature was developed for controlled drug delivery [121]. The gelling temperature of CFA dramatically decreased to 26.7 °C from 65 °C. CFA gel can be used for the sustained release of doxorubicin via the inclusion complexation of β-cyclodextrin, as well as for the complete release of bovine serum albumin. Agarose-based hybrid hydrogels with tunable, charge-selective permeability properties were prepared by mixing a series of (bio-)macromolecules with agarose [122]. These hybrid hydrogels can selectively retard the diffusion and translocation of positively or negatively charged dextran at both acidic and neutral pH, according to the type and concentration of the incorporated macromolecules. What is more, the agarose matrix provides a high level of versatility, and their permeability profiles can be tailored by integrating macromolecules with the desired physicochemical properties. Human-derived cytokine functionalized sericin/agarose hybrid hydrogels based on the hydrogen bond network structure of sericin and agarose were fabricated [123]. The hybrid hydrogels showed improved stability and mechanical performance and improved the cell-proliferation activity, suggesting a promising functional biomaterial for application in the clinic. A triple-network carboxymethyl chitosan/sodium carboxymethyl cellulose/agarose hydrogel containing silk fibroin/polydopamine nanoparticles was fabricated as an antibacterial dressing [124]. The hydrogel showed effective inhibition in the growth of *Pseudomonas aeruginosa* and supported the proliferation and growth of the fibroblast cells, suggesting a promising material to treat wound infection.

### 2.9. Dextran and Dextran-Based Hydrogel

**Figure 9:**
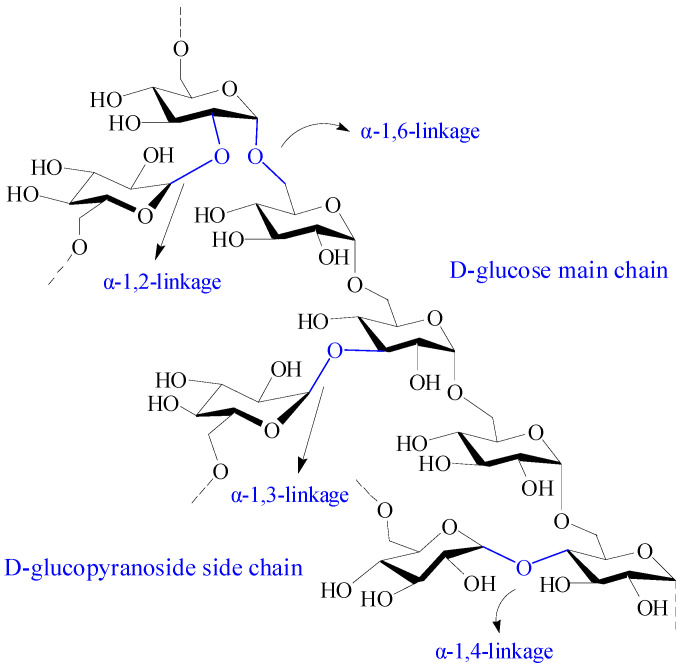
Chemical structure of dextran.

Dextran is a branched neutral glucan composed of α-1, 6 glycosidic linkages between glucose monomers, with branches from α-1, 2, α-1, 3, and α-1, 4 linkages (Figure 9) [18]. It has excellent solubility, biocompatibility, biodegradability, and nonimmunogenicity, making dextran a suitable material in biomedicine [19]. Dextran is physiologically harmless as it can be depolymerized by digestive enzymes in the lumen of the body, such as the large intestine, liver, spleen, and kidney. Moreover, dextran is an excellent starting biopolymer for structural design. It has a narrow molecular weight distribution and abundant active hydroxyl groups, which is advantageous for chemical modification. Various dextran derivatives can be prepared by reactions including esterification, etherification, and oxidation [125].

Dextran was first discovered by Louis Pasteur in 1861 from slime-producing bacteria called *Leuconostoc Lesenteroides*. Subsequently, researchers found that several Gram-positive, facultative anaerobic bacteria, such as *Leuconostoc* and *Streptococcus* strains, can also produce dextran [126]. The extracellular catalyzation of D-glucopyranyl residues from sucrose to dextran by several lactobacilli via dextransucrase is the main source of dextran in nature [18]. The degree of branching units in the 2, 3, and 4 positions are related to the dextran-producing bacterial strain. In addition, dextran can be synthesized by the cationic ring-opening polymerization of levoglucosan, which is a pyrolysis product of polyglucans.

Dextran-based hydrogels have been widely used in biomedicine because of their good mechanical strength and swelling degradation properties. The methods for preparing dextran hydrogels include crystallization, physical cross-linking, chemical cross-linking, radiation cross-linking copolymerization, and so on [127]. Bioresponsive diselenide-functionalized dextran-based hydrogels with GPx-like catalytic activity was developed via Schiff base cross-linking for GSH consumption-enhanced local starvation- and hypoxia-activated melanoma therapy [128]. The dextran-based hydrogel is pH- and dual redox-responsive and could degrade sensitively and excite drug release with the increasing acid and H_2_O_2_, resulting in remarkably enhanced local anticancer efficacy. Gallic acid modified chitosan/oxidized dextran hydrogels were prepared via Schiff base cross-linking for wound healing [129]. The hydrogel showed an excellent self-healing ability, good injectability, strong antioxidant activity, favorable biocompatibility, and accelerated wound healing through the elimination of ROS in burn injury.

## 3. Natural Polymer-Based Hydrogels for Biomedical Applications

Hydrogels based on natural polymers have emerged as promising alternatives for the ECM in biomedical applications due to their unique integration of biodegradability, biocompatibility, mechanical property tunability, biomimicry, and responsiveness, which could provide microenvironments for the preservation of cellular functions, promotion of cell health, and encouragement of tissue formation [9]. A variety of hydrogels formulated using natural biopolymers have been fabricated as drug carriers, cell delivery systems, scaffolds for tissue engineering, and so on, which provide versatile platforms for medicinal applications.

### 3.1. Drug Carriers for Drug Delivery

Hydrogels represent a class of drug delivery systems that have excellent drug delivery performance. The entangled polymer networks of the hydrogels can trap a large amount of water as well as hydrophilic small/macromolecular drugs without dissolving, protecting the drug molecules from hostile environments, such as enzymes and low pH values [130]. In addition, smart hydrogels containing sensor moiety that can response to various physical (i.e., temperature, sound, electric and magnetic fields) and/or chemical stimuli (i.e., pH, oxygen concentration, enzyme, glucose, and specific molecular recognition events) can undergo reversible gel–sol phase or volume phase transitions upon minute even second change in the environmental condition, making them ideal candidates for controlled drug release systems [131]. With the wide application of hydrogels in drug delivery, the demand for hydrogels is increasing, and hydrogels have been designed to possess various functions, such as conductivity, self-healing ability, and so on. Besides the hydrogels prepared by natural polymer only, multicomponent hydrogel materials prepared by natural polymers integrated with semisynthetic/synthetic polymers and/or inorganic particles often exhibit a variety of functions. Several excellent reviews of intelligent hydrogels for drug delivery based on synthetic hydrophilic polymers have been reported previously [132], and hydrogels based on chitosan as drug carriers have also been reviewed elsewhere [133]. In this part of the review, the recent process of natural polymer-based hydrogels for drug delivery is outlined, including the composite hydrogels formed from a natural polymer combined with a synthetic polymer or inorganic nanoparticles (Table 2).

Chitosan-based micellar hydrogels with self-healing and injectable properties were developed from phenolic chitosan and a micellar cross-linker for stroke therapy [134]. Minocycline (a hydrophilic drug) and edaravone (a hydrophobic drug) were individually encapsulated in the hydrophilic network and hydrophobic micellar core of the hydrogel and exhibited an asynchronous releasing behavior with a first-order rapid release for minocycline and a zero-order sustained release for edaravone. Rats treated with the dual-drug-loaded hydrogel showed a behavioral improvement with ≈84% recovery and a balanced brain midline shift with ≈0.98 left/right hemibrain ratio. Leach et al. developed a multidomain peptide hydrogel for cyclic dinucleotide (CDN) delivery [135]. Compared to standard collagen hydrogel, that multidomain hydrogel exhibited an eightfold slower release rate of CDN and a sixfold improvement in survival of mice. A chitosan/poly (glutamic acid)/alginate polyelectrolyte complex hydrogel with a homogeneous structure and excellent performance was prepared via a newly developed semidissolution/acidification/sol–gel transition method [136]. Piroxicam was in situ embedded in the hydrogel successfully and showed a controlled colon-specific drug release, reducing the gastrointestinal irritation side effect of piroxicam. Mukhopadhyay and coworkers developed a precisely controlled host–guest interaction system to accomplish a controlled stepwise release and capture of the guest molecule (cyclodextrin) using a coordinated polymer-based host with an azobenzene side appendage and temperature as stimuli [137], exhibiting a temperature-responsive stepwise release of α-CD both in the solution and quasi-solid (hydrogel) states.

Magnetic nanoparticles with a low toxicity and biocompatibility, like Fe_3_O_4_ particles, are commonly introduced into drug delivery systems, rendering the drug carrier with a targeted and controlled delivery character [139,143]. Novel magnetic hydrogel microrobots featured with a sustained drug release, targeted movement, satisfactory antioxidant properties, and biosafety were developed based on alpha-lipoic acid conjugated gelatin methacryloyl for inner ear administration [138]. The pre-injection of the magnetic hydrogel microrobot into the middle ear of cisplatin-deafened mice could effectively prevent deafness. Kesavan et al. reported hybrid gel beads based on chitosan and Fe_3_O_4_ cross-linked polyethylene glycol for rifampicin (an antituberculosis drug) delivery [139]. The magnetic gel beads were a pH- and magnetic field-responsive asset in the drug delivery application. CaCO_3_/sodium alginate/Fe_3_O_4_ hydrogel-based capsule microrobots were prepared by a triaxial microfluidic chip for intravascular targeted drug delivery [140]. After intravenous injection, the hydrogel-based capsule microrobots could reach the destination in blood vessels along the predetermined trajectory through an external magnetic field.

Carbon quantum dots are another extensively investigated inorganic material for drug delivery owing to their excellent properties, for instance, their chemical inertness, great dispersibility, easy changeability of size and shape, high surface area with delocalized electrons, intensive fundamental fluorescence, unequaled excitation relevant emission, local functional groups at the edges, and drug loading capacity by π–π interactions [144,145]. Javanbakht et al. reported a novel hydrogel nanocomposite films via the incorporation of graphene quantum dot in to carboxymethyl cellulose hydrogel for doxorubicin delivery [141]. The nanocomposite hydrogel films showed a pH-sensitive and consecutive prolonged release of doxorubicin, and nonobvious cytotoxicity on K562 cells. Pooresmaeil et al. developed pH-sensitive bionanogels via chemical the cross-linking of carbon dots and gelatin using dialdehyde carboxymethyl cellulose for both bioimaging and tumor-targeted codrug delivery [142]. Curcumin and doxorubicin were loaded in the nanogels with a drug entrapment efficiency of ~44.0% and 41.4%, respectively. The bionanogels showed a pH-controlled release for both drugs and a superior anticancer effect in comparison with free curcumin/doxorubicin.

### 3.2. Wound Dressings for Wound Healing

In recent years, hydrogels have aroused great interest as wound dressing owing to the high content of water in the three-dimensional hydrophilic polymeric networks, which could afford a moist environment for the wound site, cooling the surface of lesion skin, alleviating the pain, helping wounds to heal faster [146,147]. In addition, hydrogels with porous and continuous networks are capable of absorbing wound fluids, facilitating the exchange of nutrients and metabolites, adjusting the oxygen concentration, and protecting the wound from infection [148]. However, wound healing is a complicated process comprising several overlapping stages, such as hemostasis, inflammation, proliferation, re-epithelialization, and remodeling [149], which involves the systematic, coordinated, and balanced activity of inflammation, vascular blood, connective tissue, and epithelial cells. In this part of the review, the recent process of natural polymer-based hydrogels for wound healing is outlined (Table 3).

Sodium alginate–polyacrylamide hydrogels were prepared for antibacterial study and wound healing using a novel cross-linking strategy by divalent ions, such as copper, zinc, strontium, and calcium [150]. The results indicated that hydrogels cross-linked by zinc had good antibacterial activities and could enhance wound healing by promoting fibroblasts migration, vascularization, collagen deposition, and granulation tissue formation. It has been reported that calcium cross-linked hydrogels are preferable for clinical applications. For instance, alginate dressings are best suited for moist wounds. The sodium in the wound exudate undergoes an ion exchange with the calcium from the alginate, and the generation of a free calcium ion can amplify the coagulation cascade, making the dressing exhibit remarkable hemostatic properties [69]. Antibacterial hydrogels with conductive, adhesive, and self-healing properties are prepared based on oxidized sodium alginate grafted dopamine/carboxymethyl chitosan/Fe^3+^ via Schiff base and Fe^3+^ coordination bonds [151]. The hydrogels showed photothermal antibacterial properties under near-infrared irradiation and improved the wound repair in the infected skin wound in mice by reducing inflammation and increasing vascular regeneration. Yang and coworkers developed an intelligent responsive MXene-based hydrogel system as photo- and magnetic-responsive drug delivery carrier for deep chronic wound healing [152]. The system integrated a dual-network hydrogel composed of covalently cross-linked poly(N-isopropyl acrylamide) and ionically cross-linked alginate with MXene-wrapped Fe_3_O_4_@SiO_2_ magnetic nanoparticles. The hydrogel system exhibited a precise control release of AgNPs when exposed to an alternating magnetic field or near-infrared irradiation and presented desirable performance in chronic diabetes wound healing by eliminating attached bacteria and promoting M2 macrophages’ polarization and angiogenesis. A novel epsilon-polylysine modified cellulose/γ-PGA double-network hydrogel with good biocompatibility and antibacterial activity was designed for wound repair [153]. The hydrogel exhibited a promoted wound healing effect in infected skin wounds by improving collagen deposition, accelerating vascularization, and enhancing cell proliferation.

Biomolecules including enzymes, proteins, proinflammatory factors, and angiogenic markers have been reported to play a vital role in the successful healing of wounds [157]. Thus, functionalized hydrogel formulations containing bioactive substances, such as protein drugs, growth factors, and live cells, which could upregulate or downregulate these markers, were developed for wound care, especially for nonhealing wounds such as diabetic wounds. Glucose-responsive hydrogels based on hyaluronic acid, phenylboronic acid, fulvic acid, and EN106 have been prepared via the dynamic cross-linking of phenylboronate ester for improved diabetic wound repair [154]. Fulvic acid in the hydrogel serves as a cross-linking agent and provides antibacterial and anti-inflammatory abilities, and the sustained release of EN106, which is an FEM1b-FNIP1 axis inhibitor, can ameliorate oxidative stress and improve angiogenesis, thus resulting in a promoting effect in the diabetic chronic wound healing. Composite hydrogels based on sodium alginate integrated with desferrioxamine (DFO) and bioglass were designed for diabetic wound healing [155]. The results showed a promoted expression of HIF-1α and VEGF and an enhanced vascularization at the wound sites due to the synergistic effect of bioglass and DFO. Yuan and coworkers developed composite hydrogel based on epigallocatechin-modified hyaluronic acid and tyramine-grafted humanlike collagen, and integrated with deferoxamine-loaded mesoporous polydopamine nanoparticles for diabetic wounds repair [57]. The hydrogel exhibited a prominent enhancement of angiogenesis, an excellent anti-inflammatory and bacteriostatic effect, good antioxidant and hemostatic properties, and biocompatibility, promoted the M2 macrophage polarization, and enhanced diabetic wounds’ healing. Exosome-loaded hydrogels based on α-Lipoic acid modified chitosan via photo-cross-linking have been designed for diabetic wound repair [156]. The hydrogels show a strong adhesion, photoinduced self-healing, and pH/H_2_O_2_/glucose responsiveness and enable a targeted exosome release to accelerate wound healing by regulating the wound environment, such as reducing oxidative stress, lowering blood glucose levels, and promoting angiogenesis.

### 3.3. Scaffolds for Regenerative Medicine

Hydrogels have aroused great interests as scaffolds for regenerative medicine due to their inherent ability to mimic the ECM, such as having a high content of water, their ability to engraft cell at targeted sites for suspending cells, and the promotion of cell health [158]. The high permeability of hydrogels enable the interchanges of oxygen, nutrients, and soluble metabolites, rendering them as ideal ECM substitutes for tissue engineering and regenerative medicine applications [159]. Natural polymers-based hydrogels offer a versatile platform for tissue engineering owing to their specific combinations of biocompatibility, biodegradability, biomimicry, responsiveness, and mechanical tunability to mimic the native ECM, providing spatial support, preserving cellular functions, and encouraging tissue formation [160]. Collagen/alginate/fibrin-based hydrogels have been developed and investigated for soft tissue engineering [55]. The hydrogels have a thermosensitivity and mechanical stiffness similar to those of native soft tissues, showing the enhanced osteogenic potential of human mesenchymal stem cells and improved aggregation of MIN6 β-cells with the indication of pseudoislets formation, which provides a tunable microenvironment for promising pancreas and musculoskeletal tissue engineering (Table 4).

The mechanical properties of hydrogels should be matched with those of native tissues, so that the hydrogels can provide stronger functions in repairing the injured tissues. Ifkovits et al. compared the repairing ability of methacrylate hyaluronic acid hydrogels with different moduli in a rat myocardial infarction model and demonstrated that only hydrogels with high moduli could statistically decrease the infarct area and promote functional recovery [161]. Oxidized alginate–gelatin hydrogels with stronger mechanical properties showed enhanced functional recovery in myocardial infarction repair by inducing angiogenesis and facilitating cell recruitment, compared to calcium ion cross-linked alginate hydrogels [162]. Composite hydrogels incorporating relevant inorganic fillers, such as bioactive ceramics, clays, and grapheme oxide, have been reported to improve their physicochemical and biological performance in the application of tissue engineering [163]. Multifunctional composite hydrogels based on methacrylated poly(glutamic acid), methacrylated gelatin, and fibroblast activation protein inhibitor (FAPi)-loaded MnO_2_-coated calcium phosphate microspheres were prepared for osteoporotic bone defect treatment [164]. The hydrogels could effectively alleviate intracellular ROS, promote M2 macrophages polarization, and reduce inflammation in in vitro experiments and promoted the repair of osteoporotic bone defects by rescuing the ROS microenvironment and guiding the immune response in bilateral OVX-induced osteoporotic rats. RGD-modified alginate-based osteoconductive hydrogels were prepared for mesenchymal stem cell (MSC) delivery in craniofacial bone tissue engineering, and hydroxyapatite microparticles were incorporated into MSC aggregates to induce osteogenesis [165]. The hydrogels showed tunable mechanical properties and biodegradability, resulting in complete bone regeneration around ailing dental implants with peri-implant bone loss in a rat model.

In general, cells encapsulated in a three-dimensional hydrogel have an isotropic arrangement. However, many biological tissues, such as skeletal muscle, tendons, ligaments, and cardiac tissue, have an anisotropic cell arrangement. Furthermore, some physiological functions also require a cellular anisotropy, like uniaxial muscle contraction, which requires a coaxial alignment of muscle fibers. Therefore, anisotropic cell encapsulation in 3D hydrogels is highly desirable and necessary for tissue regeneration [166]. GelMA-based hydrogel scaffolds containing anisotropic microchannels were developed by a vertical 3D cryo-bioprinting technique, and live cells were encapsulated at high viability levels in desired cellular alignments to fabricate a muscle–tendon unit and a muscle–microvascular unit [167]. The hydrogel had an improved robustness and versatility and extended broad utilities in tissue engineering, especially those anisotropic in nature. Magneto-patterned cellular hydrogels were developed based on methacrylated hyaluronic acid via photo-cross-linking to generate heterogeneous tissues [168]. Diamagnetic objects, including living cells, polystyrene beads, and drug delivery microcapsules could be prepositioned in the 3D hydrogels. Engineered cartilage constructs similar to natural tissue were fabricated based on the patterned cellular hydrogels, with a high cellularity in the top region and a low cellularity in the bottom region. After a long-term culture, the cartilage constructs retained these cell gradients and subsequently created gradients in the extracellular matrix content, which enabled engineers to design and manufacture complex tissues.

### 3.4. Other Biomedical Applications

Drug discovery is of great importance in the chemotherapy of critical disease. A systematic and rapid analysis of the binding signals of small molecule drugs versus their target proteins is critical in the study of drug discovery and drug repositioning or repurposing [169]. Zhou and coworkers developed a 3D dextran hydrogel chip for the label-free and high-throughput detection of small molecule drugs [170]. They combined surface plasmon resonance imaging technology with protein microarray on the surface of a hydrogel chip, resulting in good-quality and uniform binding signals for the detected drug molecules.

Cell–cell and cell–ECM interaction can influence cell behavior, for instance the response of cells to drug treatment [171]. Thus, the generation of 3D multicellular tumor spheroids (3D TSs) is of great importance for the characterization of anticancer drugs because those 3D TSs can provide a more representative model than a conventional 2D monolayer culture, and the tumor cell–ECM interaction in a 3D culture environment is more parallel to a tumor in vivo than that in a 2D environment. Karamikamkar et al. developed 3D hydrogel beads containing alginate and collagen as in vitro tumor spheroid models using a flow-focusing process combined with an ultrasound treatment [172]. Breast cancer cells MCF-7 were encapsulated in the hydrogel beads and retained their cellular viability and proliferation ability to produce homogeneous TSs. 

The physical microenvironment of stem cells significantly affects the maintenance of pluripotency, differentiation behavior, and subsequent tissue morphogenesis [173]. Studies have shown that in vivo stem cell niches are mechanically dynamic microenvironments that constantly evolve to coordinate tissue morphogenesis and homeostasis cell activity. Thus, ECM modeling/remodeling is indispensable in regulating stem cell behavior. Various types of scaffolds, especially hydrogel systems, have been widely used in the three-dimensional microenvironment of the ECM in vivo due to their physicochemical similarities [174]. Magneto-responsive GelMA-based hydrogels were developed by incorporating Fe_3_O_4_@SiO_2_ magnetic nanorods [175]. The prealignment of nanorods under a low magnetic field enabled a dynamically and reversibly modulated modulus of the hydrogel, and the control of the hydrogel stiffness significantly influenced the differentiation of human-induced pluripotent stem cells by regulating the activation of mechano-sensitive signaling mediators. Dual-cross-linked alginate hydrogels with 3D micropatterns were prepared for the growth factors’ immobilization and stem cells’ encapsulation by photo-cross-linking of various geometries and micropattern sizes on heparin substrates [176]. Stem cells within the 3D hydrogels showed a spatially localized growth and differentiated responses to diverse growth factor patterns, indicating that the micropatterned hydrogel is a promising platform for the control of stem cell behavior in tissue engineering.

## 4. Future Perspectives

Natural polymers purified from animal or plant sources have aroused great interest in recent years because of their intrinsic properties such as chemical stability, structural versatility, good flexibility, and biocompatibility, displaying enormous potential in biomedical fields including drug delivery and tissue regeneration. Most natural polymers require complex purification or extraction processes, and the property of materials may be different according to their sources. Thus, various technologies have been explored to improve their structural properties. For example, enzymatic technology has been developed to produce hyaluronic acid with a high molecular weight, defined chain length, and low polydispersity, and biological technology such as recombinant systems has been used for the synthesis of collagen molecules [12,177]. In addition, natural polymer derivatives prepared by chemical modification combine the merits of natural polymers and synthesis polymers and have been widely used in diverse biomedical areas. What is more, the development of new natural-based polymers with advanced properties in structure and interaction with surrounding cells and tissues to achieve a promoted in vivo performance in biomedical application is still very important and challenging.

Although hydrogels based on natural polymers are attractive in biomedical application due to their bioavailability, biodegradability, biocompatibility, nontoxicity, and immunogenicity, such materials suffer from several limitations involving poor mechanical strength. Hence, various strategies have been designed to obtain strong and tough hydrogels, such as nanocomposite hydrogel materials, double-network hydrogel, and dual-cross-linking hydrogel. Nanocomposite hydrogels use micro- or nanofiller reinforcement technique to solve the limitation, and carbon materials, clays, inorganic nanoparticles, nanofibrillated cellulose, and many other inorganic materials are applied to combine with polymeric gels for the fabrication of nanocomposite hydrogels with multiple network structures and high mechanical performance [178,179]. For example, a chitosan-based nonocomposite hydrogel with a multistructural network was designed to enhance the mechanical strength by filling it with grapheme oxide (GO) nanosheets [180]. A small amount of GO filling (≤0.30 wt%) can promote the formation of multiple network structures of the hydrogel composites due to the self-assembled GO-chitosan units, thereby enhancing the mechanical properties. Double-network hydrogels based on two interpenetrating cross-linked polymer networks exhibit excellent mechanical properties due to the strong network entanglement and has seen a significant development since it was first proposed by Gong et al. [181]. Dual-crosslinking hydrogels including noncovalent and covalent bonding provide another efficient way to enhance the mechanical strength of hydrogels. For example, Chen et al. developed a dual-crosslinked hyaluronan hydrogel through the combination of electrostatic interaction and Schiff based bonding [182]. The hydrogel was further photopolymerized to achieve a high modulus and stability, exhibiting a rapid gelation and good injectability for stem cell protection with a cell viability of 92% after extrusion. 

On the other hand, various novel hydrogel materials with designed properties have been developed for biomedical applications. Intelligent hydrogels, also known as environmentally responsive hydrogels, are prepared by introducing environmental-response moieties into polymer chains, which can respond to external and/or internal stimuli and function as sensors or effectors. Many chemical and/or physical stimuli (such as temperature, pH value, light, pressure, sound, electric and magnetic fields, solvent components, ions, and some special recognition molecules) can induce the responsiveness of intelligent hydrogels. Due to the differences between the physiological microenvironment of many lesion sites and normal tissues, intelligent hydrogels have great advantages for smart drug delivery, which can target drug delivery to the lesion sites, reduce the toxicity and side effects of drugs, and improve their bioavailability. Injectable hydrogels are another new type of hydrogel materials, generally cross-linked by dynamic bonds, which can be implanted into the human body without surgery. Biomolecules and/or cells can be encapsulated in hydrogels in situ by mixing them in a polymeric solution before injection. By injection, cell-laden scaffolds can be implanted into the desired location, avoiding the pain and complexity of surgical implantation, and in situ cell fixation is also beneficial to fill irregular tissue defects. Chen et al. developed a logic-based diagnostic and therapeutic multiresponsive hydrogel to orchestrate diabetic bone regeneration [183]. The regeneration of diabetic bone defects is seriously impaired by the fluctuation of glucose level, high concentration of active oxygen species, and overexpression of proteinases. The injectable multiresponsive hydrogel with a double network consisted of phenylboronic acid crosslinked poly(vinylalcohol) and gelatin colloids, demonstrating a logic-based cargo release and the regulation of macrophage polarization for a better diabetic bone regeneration. In addition, metal organic framework (MOF)–hydrogel composites, as an emerging class of hybrid materials, have bright application prospects in drug delivery, biosensing, biocatalysis, and wound healing, owing to the combination of the functional characteristics of the two materials [184]. MOFs have sparked widespread interest in the field of chemistry and materials due to their outstanding merits of a high porosity, large surface area, tunable pore size, abundant structure, and customizable design. However, the application of MOFs is severely limited due to their instability, intrinsic fragility, poor processability, and recoverability [185]. Owing to the coordination between the side groups of polymer chains and metal ions, the inclusion of MOFs into the three-dimensional network structure of the hydrogel cannot only improve the mechanical properties and surface area of the hydrogels but also overcome the shortcomings of instability and brittleness of MOFs, and promote their stability and processability [186]. Yao et al. developed an omniphobic Zn-MOF-hydrogel composites by a microfluidic-emulsion-templating method for wound healing [187]. The hydrogel membrane with its reentrant architecture combined the advantages of hydrophilic and hydrophobic dressings, which could inhibit bacteria invasion, enabled the encapsulation and controlled release of antibacterial ingredients, and accelerated wound healing. A bioinspired synergistic antibacterial MOF–hydrogel composite for anti-infection therapy was fabricated based on reduced polydopamine nanoparticles-doped Cu-MOF and Schiff base cross-linked dodecyl chitosan-oxidized sodium alginate hydrogel [188]. The hydrogel matrix mimicked a “spider web” and immobilized bacteria, while Cu-MOF mimicked “spider fangs” and released singlet oxygen to damage bacterial outer membranes.

Furthermore, various new technologies have been explored to fabricate advanced hydrogel materials with designed properties for biomedical applications. In recent years, with the widespread usage of 3D printing, this technology has been adopted to create cell-laden hydrogels with biomimetic structures for regenerative medicine and tissue engineering. This technique of 3D printing living cells is also known as 3D bioprinting. Due to the attractive features, such as a high water content and being highly porous and permeable, which mimics the ECM and helps cell attachment and migration, hydrogels are ideal candidates for bioinks as both cellular carriers and structural constituents [189]. The most frequently used 3D bioprinting techniques include extrusion, injection, laser-assisted bioprinting, and stereolithography. As bioinks, polymers should be highly printable to provide a robust and cell-friendly microenvironment to be both cell carriers and structure components. During the 3D bioprinting process, a polymer is transformed into a 3D structure via cross-linking, and it should ideally be in the gel phase both in the syringe and after printing, called “dual-stage cross-linked”. The first-stage cross-linking (already present in the syringe) defines the material’s rheological behavior during printing, while the second-stage cross-linking (curing step after printing) determines the final mechanical properties [190]. Wang et al. designed an implantable gelatin-based hydrogel scaffold with a porous structure loaded with immunoregulators for cancer vaccine delivery therapy using 3D printing technology [191]. Compared with a conventional hydrogel, that 3D-printed hydrogel scaffold with specific structure could recruit immune cells and inhibit tumor growth more efficiently. Zhang et al. developed a unique shrinking printing technique to enhance the resolution of 3D printing [192]. They found that by immersing a 3D-printed patterned hydrogel with network formed by polyionic polymers into a polyionic solution with opposite net charges, shrinkage can occur rapidly due to complex condensation and water expulsion, resulting in printed constructs with reduced linear dimensions compared to the original pattern. Zhang et al. developed a fast hydrogel stereolithography printing method to create a centimeter-sized, multiscale solid cell-laden hydrogel model within minutes [193]. The rapid 3D printing of large-size hydrogel models is significant to reduce the part’s deformation and cellular injury in conventional 3D printing methods. An inherent limitation of 3D printing is that the printed object is static and therefore inappropriate for dynamic remodeling in response to external stimuli. Thus, 4D printing has been created to represent a careful combination of additive manufacturing, smart materials, and appropriate geometry, which allows for an animated structure, changing its shape, function, or performance over time when subjected to specific external stimuli after fabrication [194,195]. Wang et al. developed a multiresponsive bilayer membrane comprising a hydrogel layer and a microstructured shape memory polymer layer using 4D printing technology [196]. The membrane allowed active topography changes via external heating and could tailor the mechanical property to match the demands at different stages in the bone-repair process, improving the overall efficiency.

## 5. Conclusions

Natural polymer-based hydrogels are attractive in biomedical fields due to their unique and excellent properties. The polymeric structure and process/synthesis of commonly used natural polymer for hydrogel preparation and the natural polymer-based hydrogels including hydrogel formation and property were elaborated. The biomedical applications of hydrogels based on natural polymers in drug delivery, tissue regeneration, wound healing, and other biomedical fields like drug discovery, cell–cell and cell–ECM interaction, and cell guiding were summarized. With the development of biomedicine, hydrogels as well as polymers with a high performance are highly desirable. Thus, new technologies such as enzymatic and biological methods are developed to improve the structural properties of natural polymer and to develop novel natural-based polymers or natural polymer derivatives with high performance. In addition, novel hydrogel materials (such as hybrid and/or composite hydrogels, intelligent hydrogels with stimuli responsiveness, and injectable hydrogels) are designed to meet the advanced requirements of biomedical applications, and new strategies (such as in situ cross-linking, dual-cross-linking, and 3D/4D bioprinting) have been explored to fabricate advanced hydrogel materials with design properties for biomedical applications.

## Figures and Tables

**Table 1:** Natural polymer used for hydrogels’ formation.

Natural Polymer	Chemical Structures	Preparation and Processing	Ref.
Cellulose	Composed of β (1-4)-glycosidic-linked glucose units	1. Lignocelluloses purification by chemical treatment. 2. Biological method depending on microbial enzymes. 3. Bacterial cellulose produced by certain types of bacteria.	[9]
Chitosan	Poly-(β-1-4) N-acetyl-D-glucosamine	Derived from chitin by partial deacetylation though chemical or enzymatic hydrolysis.	[10,11]
Collagen	A helical fibrous protein formed by three peptide chains	1. Extracted and purified from various animal sources by chemical and enzyme treatment. 2. Recombinant collagen produced by recombinant technology and biosynthesis.	[12]
Alginate	Consisting of α-L glucuronate and β-D mannuronate repeating units	1. Extraction from brown algae (Phaeophyceae) by treatment with aqueous alkali solutions. 2. Bacterial biosynthesis from Azotobacter and Pseudomonas.	[13]
Hyaluronic acid	Consisting of N-acetyl-glucosamine and D-glucuronic acid residues	1. Extraction from animal tissues. 2. Microbial fermentation using pathogenic bacteria and nonpathogenic bacteria. 3. Enzymatic polymerization of UDP-sugar monomers.	[14]
Starch	Composed of α-D-(1-4) and α-D-(1 - 6)-glycosidic-linked glucose units	Extracted from seeds, roots, tubers, stems, fruits, and all leaves.	[15]
Guar gum	Composed of (1-4)-β-D-mannopyranosyl units and (1-6)-α-D-galactopyranosyl units	Isolated from the embryos of the leguminous plant *Cyamopsis tetragonoloba*.	[16]
Agarose	Consisting of alternating 1,3-linked β-D-galactose and 1,4-linked 3,6-anhydro-α-Lgalactose units	Extracted from seaweed.	[17]
Dextran	Composed of α-1, 6 glycosidic linkages between glucose monomers, with branches from α-1, 2, α-1, 3, and α-1, 4 linkages	1. Produced from slime-producing bacteria called *Leuconostoc Lesenteroides;* 2. Produced from several Gram-positive, facultative anaerobic bacteria, such as *Leuconostoc and Streptococcus* strains; 3. Synthesized by cationic ring-opening polymerization of levoglucosan.	[18,19]

**Table 2:** Natural polymer-based hydrogels as drug carriers for drug delivery.

Hydrogels	Drugs	Properties and Function	Ref.
Chitosan-based micellar hydrogels	Minocycline and edaravone	Self-healing and injectable property. First-order rapid release for minocycline and zero-order sustained release for edaravone. Behavioral improvement in stroke rats.	[134]
Multidomain peptide hydrogels	Cyclic dinucleotide (CDN)	An eightfold slower release rate of CDN and a sixfold improvement in survival of mice compared with standard collagen hydrogel.	[135]
Chitosan/poly (glutamic acid)/alginate polyelectrolyte complex hydrogels	Piroxicam	Controlled colon-specific drug release and reduced gastrointestinal irritation side effect of piroxicam.	[136]
Host–guest interaction hydrogel system	α-CD	Temperature-responsive stepwise release of α-CD both in the solution and hydrogel states.	[137]
Magnetic hydrogel microrobots	Alpha-lipoic acid	Sustained drug release, targeted movement, satisfactory antioxidant properties and biosafety.	[138]
Hybrid gel beads based on chitosan and Fe_3_O_4_ cross-linked polyethylene glycol	Rifampicin	pH- and magnetic field-responsive asset in drug delivery.	[139]
CaCO_3_/sodium alginate/Fe_3_O_4_ hydrogel-based capsule microrobots	Indomethacin	Intravascular targeted drug delivery by following a predetermined trajectory in the blood vessel under magnetic drive.	[140]
Grapheme quantum dot/carboxymethyl cellulose-based hydrogel nanocomposite films	Doxorubicin	pH-sensitive and consecutive prolonged release of doxorubicin and nonobvious cytotoxicity on K562 cells.	[141]
Carbon dots/gelatin/carboxymethyl cellulose-based bionanogels	Curcumin and doxorubicin	pH-controlled release for both drugs and superior anticancer effect in comparison with free curcumin/doxorubicin.	[142]

**Table 3:** Natural polymer-based hydrogels as wound dressings for wound healing.

Hydrogels	Properties	Effects in the Wound Healing	Ref.
Sodium alginate–polyacrylamide hydrogels	Excellent mechanical strength by zinc cross-linked hydrogel.	Antibacterial activities and promoted fibroblasts migration, vascularization, collagen deposition, and granulation tissue formation.	[150]
Alginate/dopamine/carboxymethyl chitosan-based hydrogels	Antibacterial, conductive, adhesive, and self-healing properties.	Photothermal antibacterial property, reduced inflammation, and increased vascular regeneration.	[151]
Alginate/MXene-based hydrogel	Photo- and magnetic-responsive, and precise control release of AgNPs.	Eliminated bacteria attachment and promoted M2 macrophages polarization and angiogenesis.	[152]
Epsilon-polylysine modified cellulose/γ-PGA double-network hydrogel	Good biocompatibility and antibacterial activity.	Improved collagen deposition, accelerated vascularization, and enhanced cell proliferation.	[153]
Hyaluronic acid-EN106 hydrogels	Glucose-responsive, antibacterial and anti-inflammatory abilities, and sustained release of EN106.	Ameliorated oxidative stress and improved angiogenesis.	[154]
Sodium alginate hydrogel containing desferrioxamine (DFO) and bioglass	Injectable and sustained release of DFO.	Promoted wound healing by increasing HIF-1α and VEGF expression and vascularization.	[155]
Composite hydrogels based on hyaluronic acid/collagen/deferoxamine loaded polydopamine nanoparticles	Desirable mechanical property, improved tissue adhesive and injectable performance.	Exhibited a prominent enhancement of angiogenesis, excellent anti-inflammatory and bacteriostatic effect, promoted the M2 polarization of macrophages, and enhanced diabetic wounds’ healing.	[57]
Exosome-loaded hydrogels based on α-Lipoic acid modified chitosan	Strong adhesion, photoinduced self-healing and pH/H_2_O_2_/glucose responsiveness	Accelerate diabetic wound healing by regulating the wound environment, such as reducing oxidative stress, lowering blood glucose levels, and promoting angiogenesis.	[156]

**Table 4:** Natural polymer-based hydrogels as scaffolds for regenerative medicine.

Hydrogels	Properties	Functions	Ref.
Collagen/alginate/fibrin-based hydrogels	Thermosensitivity and mechanical stiffness similar to native soft tissues	Enhanced osteogenic potential of human mesenchymal stem cells and improved aggregation MIN6 β-cells with the indication of pseudoislets formation.	[49]
Gelatin/FAPi-loaded microspheres composite hydrogels	Antioxidant properties	Promoted repair of osteoporotic bone defects by rescuing ROS microenvironment and guiding the immune response in bilateral OVX-induced osteoporotic rats.	[114]
RGD-modified alginate-based osteoconductive hydrogels	Tunable mechanical properties and biodegradability	Complete bone regeneration around ailing dental implants with peri-implant bone loss in a rat model.	[115]
GelMA-based hydrogel scaffolds containing anisotropic microchannels	Improved robustness and versatility	Encapsulated live cells at high viability levels in desired cellular alignments to fabricate muscle–tendon unit and muscle–microvascular unit	[117]
Magneto-patterned cellular hydrogels based on methacrylated hyaluronic acid	Prepositioned diamagnetic objects in 3D hydrogels.	Fabricated cartilage constructs similar to natural tissue with gradient cellularity and maintained these cell gradients in the extracellular matrix content.	[118]

## Data Availability

Data sharing not applicable.
